# Integrative regression network for genomic association study

**DOI:** 10.1186/s12920-016-0192-7

**Published:** 2016-08-12

**Authors:** Reddy Rani Vangimalla, Hyun-hwan Jeong, Kyung-Ah Sohn

**Affiliations:** Department of Information and Computer Engineering, Ajou University, Suwon, 443-749 Republic of Korea

**Keywords:** Genomic association, Sparse regression, Similarity fusion network, Integrative analysis, TCGA

## Abstract

**Background:**

The increasing availability of multiple types of genomic profiles measured from the same cancer patients has provided numerous opportunities for investigating genomic mechanisms underlying cancer. In particular, association studies of gene expression traits with respect to multi-layered genomic features are highly useful for uncovering the underlying mechanism. Conventional correlation-based association tests are limited because they are prone to revealing indirect associations. Moreover, integration of multiple types of genomic features raises another challenge.

**Methods:**

In this study, we propose a new framework for association studies called integrative regression network that identifies genomic associations on multiple high-dimensional genomic profiles by taking into account the associations between as well as within profiles. We employed high-dimensional regression techniques to first identify the associations between different genomic profiles. Based on the resulting regression coefficients, a regression network was constructed within each profile. For example, two methylation features having similar regression coefficients with respect to a number of gene expression traits are likely to be involved in the same biological process and therefore we define an edge between two methylation features in the regression network. To extract more reliable associations, multiple sparse structured regression techniques were applied and the resulting multiple networks were merged as the integrative regression network using a similarity network fusion technique.

**Results:**

Experiments were carried out using four different sparse structured regression methods on five cancer types from TCGA. The advantages and disadvantages of each regression method were also explored. We find there was large inconsistency in the results from different regression methods, which supports the need to extract the proposed integrative regression network from multiple complimentary regression techniques. Fusing multiple regression networks by using similarity measurements led to the identification of significant gene pairs and a resulting network with better topological properties.

**Conclusions:**

We developed and validated the integrative regression network scheme on multi-layered genomic profiles from TCGA. Our method facilitates identification of the strong signals as well as weaker signals by fusing information from different regression techniques. It could be extended to integrate results obtained from different cancer types as well.

**Electronic supplementary material:**

The online version of this article (doi:10.1186/s12920-016-0192-7) contains supplementary material, which is available to authorized users.

## Background

Ongoing efforts by the The Cancer Genome Atlas (TCGA) [1] or the International Cancer Genome Consortium (ICGC) [2] have provided an exceptional opportunity for biomedical researchers and practitioners to explore the mechanisms and to identify important biomarkers underlying cancer. Large-scale analysis of the available datasets that cover genomic, transcriptomic, and epigenomic, and clinical profiles have revealed important characteristics of genomic associations in cancer. Additionally, ‘cancer stat fact sheets’ have revealed new cases and the expected mortality rate of cancer are rapidly increasing [3]. Ongoing studies of gene expression with respect to multi-layered genomic features are highly useful for overcoming the poor prognosis of cancer.

In this study, we identified genomic associations using multiple genomic profiles. Given the high level of noise and extremely large data dimension, simple correlation-based association tests are prone to revealing indirect or false-positive genomic associations. Instead, we employed high-dimensional multivariate regression techniques to identify genomic associations between different high-dimensional genomic profiles. Moreover, we constructed a regression network utilizing the regression coefficient vector or matrix. The regression network was constructed within each profile such as mRNA expression or methylation, but takes into account the association between the two different genomic profiles. To extract more robust and statistically significant results, we used multiple regression techniques and then integrated the resultant regression networks into an integrative regression network by using a network fusion technique.

Various sparse structured regression techniques have been proposed to address the challenges arising in a high-dimensional regression setting, both for the input and output variables. A widely used *L*_1_-regularized linear regression known as Lasso [4] produces sparse regression coefficients when the number of features is large. Variants of Lasso have been proposed to incorporate structural information of genomic features in input and expression traits as output. Graph-guided Fused Lasso (GFLasso) [5], for example, utilizes the network structure among output variables in multiple output regression setting. This is particularly suitable for association studies that consider gene expression traits as output variables because gene expression traits are under a natural network structure. In Sparse Group Lasso (SGL) [6], input variables (genomic features) are assumed to behave in groups; thus, by utilizing grouping information of the features such as pathway groups, the method identifies important genes in common pathways of interest. For problems, such as grouped covariates, this method can impose sparse effect on the group level and within the group level. The more recently described Structured Input–output Lasso (SIOL) method combines structural constraints on both the inputs and the outputs [7]. Similar to GFLasso, this method considers output structural information, and similar to SGL, considers input group information. SIOL predicts true non-zero coefficients using both structural information and grouping effect on the inputs and output variants.

Each of these sparse structured regression methods exhibits advantages and disadvantages. Rather than selecting the single best method, we build an integrative regression network by fusing multiple regression networks. We adopted the existing approach of Similarity Network Fusion (SNF) [8] for network integration. The final fused network could compile shared information as well as complementary information from all different datasets used in the fusion by identifying similarities in all of the networks. Given the natural propagative behavior of SNF, the produced output showed less noise and captured important signals (both stronger and weaker signals). We demonstrate the proposed approach for an association study using methylation and gene expression data for five cancer datasets from TCGA.

## Methods

### Overview of the proposed method

**Fig. 1:**
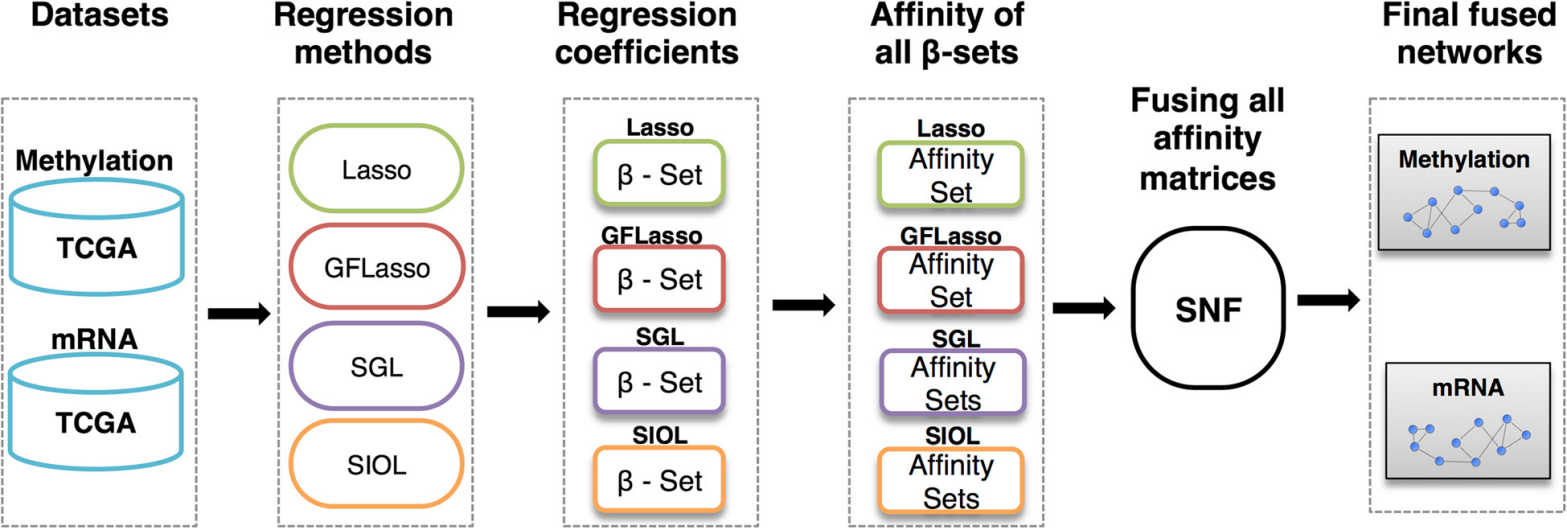
Overview of the complete workflow process

The complete flow of the process is depicted in Fig. 1. Methylation data and mRNA expression traits from TCGA were used in this study. Multiple regression methods were performed on the data by treating methylation data as features and expression traits as outputs. Resulting regression coefficients matrices were used to construct affinity or similarity networks on each profile. The constructed networks were finally fused as an integrative regression network using the similarity network fusion technique.

### Data & pre-processing

We downloaded gene expression data and methylation data used in a previous study [8] that were collected for five different cancer profiles from TCGA: glioblastoma multiforme (GBM), breast invasive carcinoma (BIC), lung squamous cell carcinoma (LSCC), kidney renal clear cell carcinoma (KRCCC), and colon adenocarcinoma (COAD). Acquisition platforms of the expression traits and methylation data are shown in Table 1 [8, 9].

**Table 1:** Data acquisition platforms

Cancer type	Expression data	Methylation data
GBM	Broad Institute HT-HG-U133A Platform	JHU-USC-Illumina-DNA-Methylation Platform
LSCC		JHU-USC-Human-Methylation-27 Platform
BIC	UNC-Agilent-G4502A-07 Platform	
COAD		
KRCCC	UNC-Illumina-Hiseq-RNASeq Platform	

The methylation features were converted into gene-based representations by taking the average values as reported previously [9]. To identify the common behavior of genes across multiple cancer profiles, the common methylation genes and expression traits across all cancer types were collected. This was done to study the collective and crucial genes in all cancer data types. This resultant final datasets contained 597 methylation features and 10,299 expression traits. We focused on cancer-related genes from COSMIC [10] and took the intersection of those genes with 10,299 expression traits of BIC, GBM, LSCC, KRCCC, and COAD. Table 2 summarizes the final datasets for all cancer types used in this experiment. The feature values of each individual dataset were standardized such that the mean of each feature was zero and the standard deviation (SD) of each feature was one, which in turn resulted in the representation of different genomic features on expression traits without bias. For ease of understanding, the cancer profile names are as follows: BIC as breast, KRCCC as kidney, GBM as GBM, LSCC as lung, and COAD as colon.

**Table 2:** Details of all cancer profiles before and after filtration process

Cancer type	Total samples	DNA Methylation		mRNA Expression	
		Before filtering	After filtering	Before filtering	After filtering
GBM	215	1,491	597	12,042	385
BIC	105	23,094		17,814	
KRCCC	124	24,532		20,532	
LSCC	105	27,578		12,042	
COAD	92	27,578		17,814	

### High-dimensional regression methods

#### Least absolute shrinkage and selection operator (Lasso)

Lasso is a sparse regression framework that is a highly effective method for detecting associations in high dimensional data with the ability for simultaneous feature selection and regression [4]. This regression method is used to identify methylation features that are associated with gene expression traits. For the available methylation features *X*_*1i*_, *X*_*2i*_, *X*_*3i*_, and *X*_*Ji,*_ (where *J* is the total number of features and *i* is the index of the samples), the effect to *Y*_i_, the expression level of a given gene in sample *i* is modeled in a multivariate liner regression setting as follows:

1 $$ {Y}_i={\beta}_0+{\beta}_1{X}_{1i}+{\beta}_1{X}_{2i}+\cdots {\beta}_J{X}_{Ji}+{\varepsilon}_i,\kern0.5em {\varepsilon}_i\sim N\left(0,{\sigma}^2\right) $$

The *L*_*1*_-penalized objective function is used to optimize and identify comparatively lesser genomic features that affect the expression trait.

2 $$ min{{\displaystyle \sum_i\left({Y}_i-\left({\beta}_0+{\beta}_1{X}_{1i}+{\beta}_1{X}_{2i}+\cdots {\beta}_J{X}_{Ji}\right)\right)}}^2+\lambda {\displaystyle \sum_j\left|{\beta}_j\right|} $$

The second term of equation (2) induces a sparse solution by driving many irrelevant beta coefficients to exact zeros. The result of Lasso is a set of features that are highly affined to the given expression trait and the implication power of each feature *j* is given by its regression coefficient *β*_*j*_, which provides a measure of how strongly or weakly each feature influences the traits. This procedure is applied to each of the multiple gene expression traits independently. Lasso is widely effective, when *J* (features) > > *N* (number of samples), and only a small number of inputs are expected to influence outputs. This is implemented in R using the *glmnet* package [11]. The optimal parameter *λ* was chosen by cross-validation.

#### Graph guided fused lasso (GFLasso)

GFLasso, an extension of Lasso for multiple output regression, fuses regression coefficients across correlated output variables, which is particularly suitable for analyzing gene expression traits with an inherent network structure as output traits [5]. The method includes a fusion penalty along with a Lasso penalty such that the regression coefficients across correlated traits are fused using weighted connectivity. For any feature *j*, if any two traits *m* and *l* are connected with an edge, then an additional penalty is imposed on the regression coefficients *β*_*jm*_ and *β*_*jl*_. The fusion penalty encourages the sizes of the effects *β*_*jm*_ and *β*_*jl*_ of each marker *j* on correlated traits *m* and *l* to be similar [12]. Experiments demonstrated that taking output structural information improved the sensitivity and specificity for recovering sparse structure and also increased the prediction accuracy [13]. The method deals with multiple correlated traits rather than multiple independent traits as Lasso. In equation (3), *y*_*k*_ ∈ *R*^*n*^ represents the expression levels of gene *k*, *X* ∈ *R*^*n*×*J*^ is the feature matrix, and β_k_ ∈ *R*^*J*^ is the regression coefficient vector for gene *k*, *λ* is a regularization parameter for sparsity, *γ* is a GFLasso regularization parameter and *f*(*r*_*ml*_) is the correlation between the two traits being fused. GFLasso was implemented in Matlab, with the help of the code present at ‘Sailing Lab’ [14].

3 $$ \begin{array}{l} min{\displaystyle \sum_k{\left({y}_k-X{\beta}_k\right)}^T}\left({y}_k-X{\beta}_k\right)+\lambda {\displaystyle \sum_k{\displaystyle \sum_j\left|{\beta}_{jk}\right|}}\\ {}\kern3.5em  + \gamma {\displaystyle \sum_{\left(m,l\right)\in E}f\left({r}_{ml}\right){\displaystyle \sum_j\left|{\beta}_{jm}- sign\left({r}_{ml}\right){\beta}_{jl}\right|}}\end{array} $$

To select optimal regularization parameters, we first identified the median of non-zero beta coefficients and multiplied it by the total count of gene expression traits. The obtained value was assigned to lambda as an initial value. The initial gamma was fixed as 1. The observation was carried out using different *λ* and *γ* values, for example fixing *γ* and applying different values of *λ* as *λ/2*, *λ*, and *2λ*, then fixing *λ* and changing *γ* to *γ/2*, *γ*, and *2γ*, to verify the mean squared error, regression coefficients density, and time to execute the dataset. Through empirical study, we derived lambda and gamma values as those with the smallest MSE. Based on previous studies [5], the correlation threshold was fixed as 0.7 for all datasets throughout the experiments, and thus *f*(r_ml_) was always greater than or equal to 0.7 considering only very highly correlated gene expression features.

#### Sparse group lasso (SGL)

SGL in an input-structured spare regression, which utilizes a clustering or sub-group structure in feature variables, whereas GFLasso is based on the graph structure among output variables [6]. Both SGL and GFLasso were developed based on Lasso by considering the structural information in either input or output variables. SGL is a regularized model for linear regression with *L*_*1*_ (Lasso) and *L*_*2*_ penalties that imposes sparsity both at the individual feature and group levels [15]. In equation (4), *X*^*(l)*^ is the sub-matrix of *X* with columns corresponding to the predictors in group *l*, *β*^*(l)*^ the coefficient vector of that group, and *p*_*l*_ is the length of *β*^*(l)*^. The Sparse Group Lasso can be used to identify genes that are particularly important in the pathways of interest.

4 $$ min\frac{1}{2n}\left|\right|y-{\displaystyle \sum_{l=1}^m{X}^{(l)}{\beta}^{(l)}\left|\right|{}_2^2+\left(1-\alpha \right)\lambda {\displaystyle \sum_{l=1}^m\sqrt{p_l}\left|\right|{\beta}^{(l)}\left|\right|{}_2+}}\alpha \lambda \left|\right|\beta \left|\right|{}_1 $$

Here, α ∈ [0, 1] is a parameter for convex combination of the Lasso and group Lasso penalties (α = 0 gives the group Lasso fit, α = 1 gives the Lasso fit). And *n* and *m* represents the number of samples and the number of feature groups, respectively. This is implemented in R using the ‘*SGL*’ package [16].

To define the grouping of features, we applied clustering techniques to feature data. The hierarchical clustering was chosen after observing k-means and k-median clustering techniques. The function hclust in R was used with Euclidean distance measurement and Ward’s linkage method for experiments. The number of groups was verified with different trials such as 10, 20, 50, and 80, and 20 groups was chosen because of its better clustering results and MSE. For regularization parameter selection, the minimum value of the penalty parameter, as a fraction of the maximum value, was chosen to be 0.8 and *α* was set as 0.1.

#### Structured input–output lasso (SIOL)

SIOL is a jointly structured input–output Lasso to simultaneously take advantage of both input and output structures. The method considers the occurrence of grouping effects on the inputs and outputs, which can be considered as *a priori* information [7]. Similar to GFLasso, this method considers output structural information; like SGL, this method considers group information. Experiments demonstrated that the models with either input or output structure were less effective for suppressing noisy signals, resulting in many false-positives compared to when both input and output structural information were considered [17]. SIOL can produce significantly more accurate and faster results compared to other models. Grouping structure over inputs and output groups will be available as G = {g_1_, . . . , g_|G|}_ and H = {h_1_, . . . , h_|H|_}, respectively. Group Lasso uses L_1_/L_2_ penalization to enforce that all members in each group of input/output variables are jointly relevant or irrelevant to each output/input. SIOL is formulated as in equation (5). SIOL is implemented in Matlab, with the help of the code present at ‘Sailing Lab’ [18].

5 $$ min\frac{1}{2}\left|\right|Y-BX\left|\right|{}_F^2+{\lambda}_1\left|\right|B\left|\right|{}_1+{\lambda}_2{\displaystyle \sum_{k=1}^K{\displaystyle \sum_{g\in G}\left|\right|{\beta}_k^g\left|\right|{}_2}+}{\lambda}_3{\displaystyle \sum_{j=1}^J{\displaystyle \sum_{h\in H}\left|\right|{\beta}_h^j\left|\right|{}_2}} $$

Similar to SGL, the number of clusters/groups was chosen as 20. Parameter tuning was performed individually on each dataset through cross-validation. The identified **λ**_**1**_ was 0.1, **λ**_**2**_ ranged from 0.25 to 0.35, and **λ**_**3**_ ranged from 0.15 to 0.25 for all cancer profiles.

### Construction of regression network and its integration

**Fig. 2:**
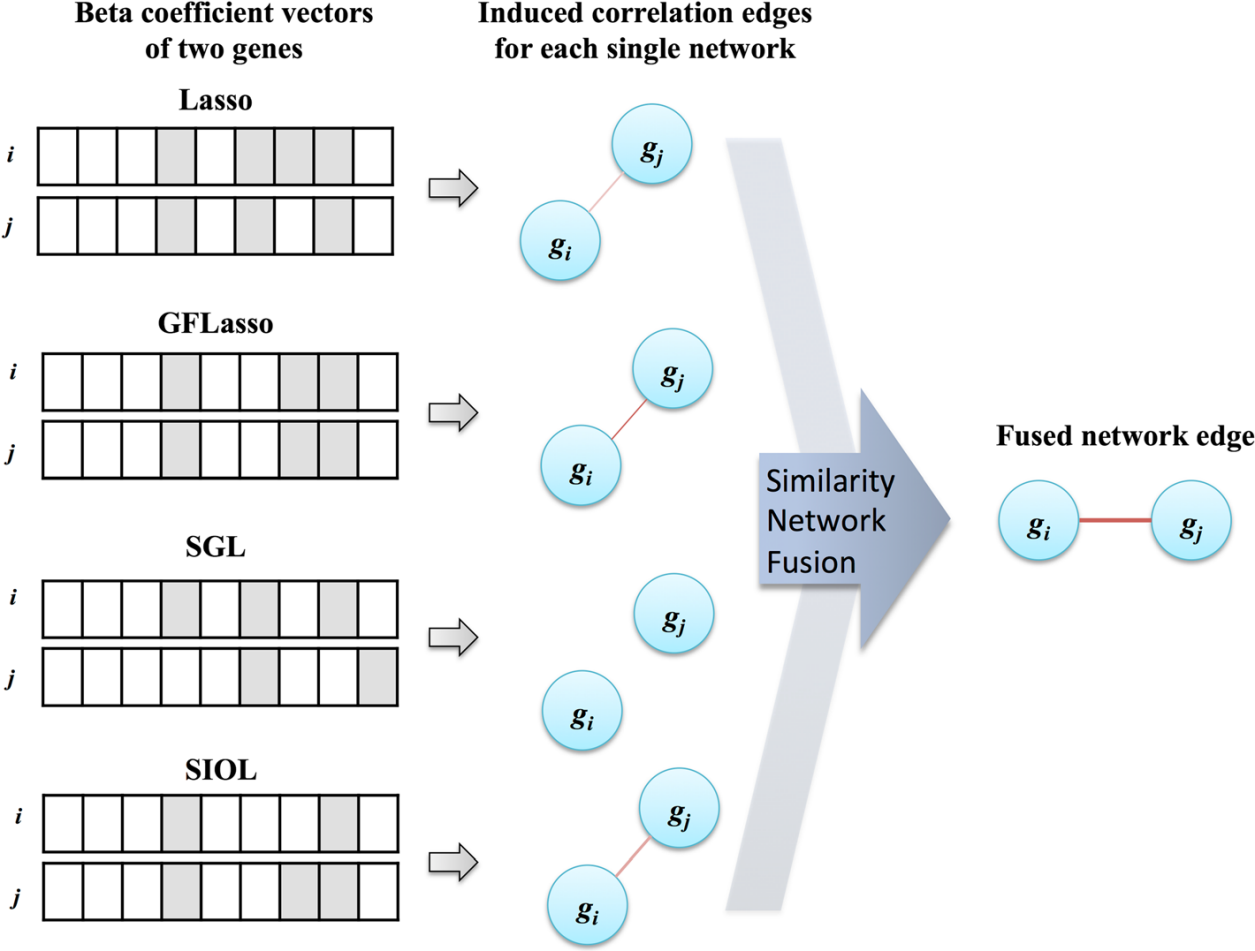
Illustration of regression network construction from regression coefficients. Two beta coefficients with similar effects for two different genes were fused using similarity measurement

Considering that two features with similar regression coefficients over expression traits have equally prominent weightage for the traits, or the traits affected by a similar set of features may be regulated by common biological processes, we constructed a regression network based on regression coefficient similarity. To obtain an unbiased result, the regression coefficient weights were normalized in SNF. Suppose that both gene ***g***_***i***_ and gene ***g***_***j***_ have similar regression coefficients with respect to the considered features as illustrated in Fig. 2. We thus defined an edge between *g*_*i*_ and *g*_*j*_. This led to a network among the output variables, in our case a gene expression network. We defined the methylation feature network using a similar idea. The edge weight was defined as the affinities of the regression coefficient vectors in SNF. In this study, to obtain more reliable true-positive signals, the results of all regression methods were combined as the *integrative regression network*, which easily revealed highly influential features, genes, and their associations. The final fused network was constructed by identifying such gene pairs, as shown in Fig. 2. SNF was mainly used for integration of different similarity networks into a single network [8]. The final fused network can be used to apprehend shared information as well as complementary information of all datasets used for fusion by observing similarities of each network. Weaker similarities disappeared in the fused network, which in turn decreased noise, while also allowing for low weighted edges supported by all networks, increasing the signal strength.

In SNF, the edge weight matrix **W** is converted into a full kernel normalized weight matrix **P** = **D**^−**1**^**W**, where **D** is the diagonal matrix whose entries ***D****(i, j) = Σ*_*j*_***W****(i, j)*. The local affinity **S** is measured using *K* nearest neighbors on the weight matrix **W**. For the given two similarity matrices in a general SNF setting, an important step is to iteratively update the matrix corresponding to each of the affinity matrices as in equation (6).

6 $$ \begin{array}{l}{P}_{t+1}^{(1)}={S}^{(1)}\times {P}_t^{(2)}\times {\left({S}^{(1)}\right)}^T\\ {}{P}_{t+1}^{(2)}={S}^{(2)}\times {P}_t^{(1)}\times {\left({S}^{(2)}\right)}^T\\ {}{P}^{(c)}=\frac{P_T^{(1)}+{P}_T^{(2)}}{2}\end{array} $$

where *P*_*t* + 1_^(1)^ is the similarity matrix for the first data type after t iterations, *P*_*t* + 1_^(2)^ is a similarity matrix of second data type, and *P*^(*c*)^ is overall status matrix. Note that the equation (6) determines the similarity fusion of two data types, and the method ensembles are equally good for more than 2 datasets. In our experiments, we used four datasets, one from each regression result, and 20 iterations (t = 20) for applying a propagating effect on the fused network. Given the propagation effects of SNF, if two nodes do not have greater similarity in one network but possess strong similarity in another network, then the pairs will be propagated by SNF to the final fusion network. SNF was implemented in R using the ‘SNFtool’ package, and both the affinity measurement and fusion techniques were supported by this package [19]. Network fusion was carried out using a non-linear method that works on message-passing theory [20]. The number of neighbors (K) and hyper parameter (alpha) are the two parameters determined by SNF. We considered the range of K to be 2–20 and for α to be 0.3–0.8. For all combinations of K and α, affinity measurement was performed using SNFtool and correlation was measured using equation (7).

7 $$ f\left(W,{w}_f\right)=\frac{{\displaystyle \sum_{i=1}^NSIM\left({w}_f,{w}_i\right)}}{N}+\frac{{\displaystyle \sum_{i=1}^{N-1}{\displaystyle \sum_{j=i+1}^N\Big(1-SIM\left({w}_i,{w}_j\right)}\Big)}}{\left(\begin{array}{c}\hfill N\hfill \\ {}\hfill 2\hfill \end{array}\right)} $$

where *N* = 4, *w*_*1*_, *w*_*2*_, *w*_*3*_, and *w*_*4*_ are the affinity sets measured using SNFtool and *w*_*f*_ is the final fused set obtained by fusing *w*_*1*_, *w*_*2*_, *w*_*3*_, and *w*_*4*_. *SIM (w*_*f*_, *w*_*i*_*)* is the correlation measurement between affinity sets *w*_*f*_ and *w*_*i*_. Finally, the highest correlation and its respective *K* and *α* for each cancer dataset were identified. Using the regression coefficient matrix as-is gives a similarity measurement of methylation features, while transposing the regression coefficient matrix gives the similarity measurement of expression traits. Using the affinity matrices and fusion techniques, we computed the optimal value of *K* and *α* for all cancer profiles.

### Identifying a cutoff for edge filtering in regression networks

A cutoff was computed to choose the gene pairs with more significance from each individual affinity matrix and from the final fused matrix as in [21]. The affinity matrix of each method was randomly permuted 100 times and a cutoff value was determined using equation (8). The identified cutoff is a point at which the total number of edges in the permuted network is less than the real network and the largest connected component is larger than any other connected component. *W* denotes a real network and *WP*_*k*_ is the *k*^th^ permutation network. E(*X*) and C(*X*) are the number of edges and largest connected components in the network *X*. In equation (8), the numerator is the average of permuted network’s total number of edges and largest connected components, while the denominator is the number of edges and largest connected components of the real network.

8 $$ \begin{array}{l}f\left(W,WP\right)= argmi{n}_{c\in \left[0,1\right]}\frac{1}{2}\left[\frac{\frac{1}{\left|WP\right|}{\displaystyle \sum_{k=1}^{\left|WP\right|}E\left(W{P}_k^c\right)}}{E\left({W}^c\right)}+\frac{\frac{1}{\left|WP\right|}{\displaystyle \sum_{k=1}^{\left|WP\right|}C\left(W{P}_k^c\right)}}{C\left({W}^c\right)}\right]\\ {}\mathrm{w}\mathrm{here}\\ {}{W}^c=\left\{\left(i,j\right)\Big|\left(i,j\right)\in W\kern0.5em \mathrm{and}\kern0.5em {\mathrm{w}}_{i,j}\ge c\right\}\\ {}W{P}_k^c=\left\{\left(i,j\right)\Big|wp{(k)}_{i,j}\in W{P}_k\kern0.5em \mathrm{and}\kern0.5em \mathrm{w}\mathrm{p}{\left(\mathrm{k}\right)}_{i,j}\ge c\right\}\end{array} $$

## Results

### Performance investigation of different regression methods

**Fig. 3:**
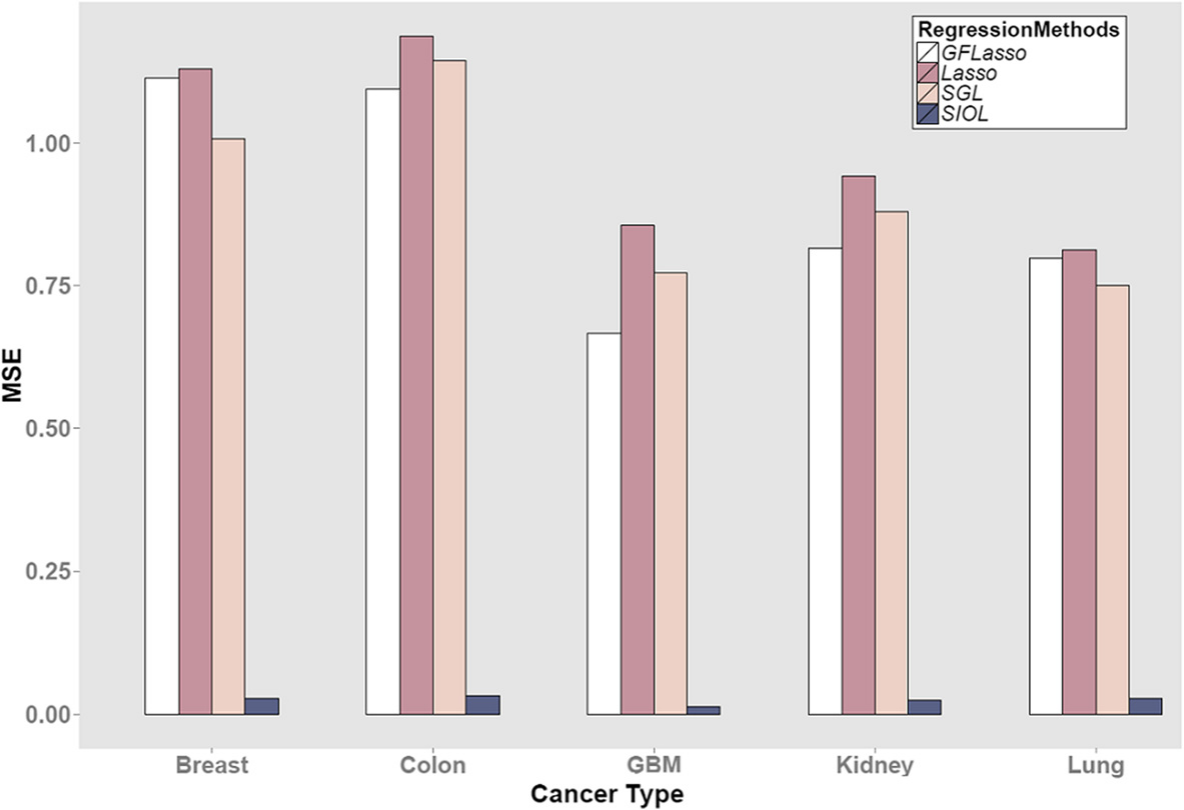
Comparison of regression methods in terms of mean squared error (MSE)

We first compared the performance of each regression method considered in this study. To evaluate each method, 2/3 of the dataset was used as training data and the remaining 1/3 was used as test data. Figure 3 shows the MSE of all methods for the five types of cancer datasets. A smaller MSE implies better performance.

Because of its structural information in consideration of SIOL, this method significantly outperformed all other regression methods, whereas GFLasso, SGL, and Lasso tend to produce comparable results, while Lasso, which uses no structural information, produces the largest MSE. The overall performance in terms of MSE in decreasing order was SIOL, GFLasso, SGL, and Lasso. The procedure was applied for multiple cancer profiles as shown in Fig. 3 and the behavior was observed to be similar for all cancer data types.

### Discovering common genomic features of all methods without fusion technique

We further investigated the combined results of all four methods to identify influential predictors of cancer. The common predictors that were retrieved using all regression methods were collected. We focused on genomic features identified using all methods, as they are the strongest predictors of the expression traits. As the *β* value is the measure of how strongly each predictor variable influences the response variable, highly impacted gene pairs (top 200) based on the *β* values were collected for each of the four regression methods.

**Fig. 4:**
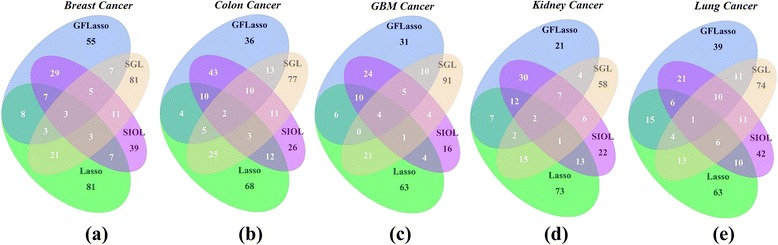
Venn diagram of methylation features associated with expression traits. Methylation features found by top 200 regression coefficients is depicted for (**a**) Breast, (**b**) Colon, (**c**) GBM, (**d**) Kidney, and (**e**) Lung cancer

Figure 4 shows the Venn diagrams for the common methylation features associated with expression traits between different regression methods. Figure 1(a) to (e) shows the result using the top 200 regression coefficients on the five cancer profiles. Overall, the number of common features is very small across all cancer types, and between any pair of regression methods. The common genomic features found using the methods GFLasso and SIOL, which showed higher values than any other combination, are 29, 43, 24, 30, and 21 for breast, colon, GBM, kidney, and lung cancer profiles, respectively. Consideration of structural information (GFLasso and SIOL) appeared to be the main cause of this behavior. The fusion penalty applied for output variants was common in both methods.

**Fig. 5:**
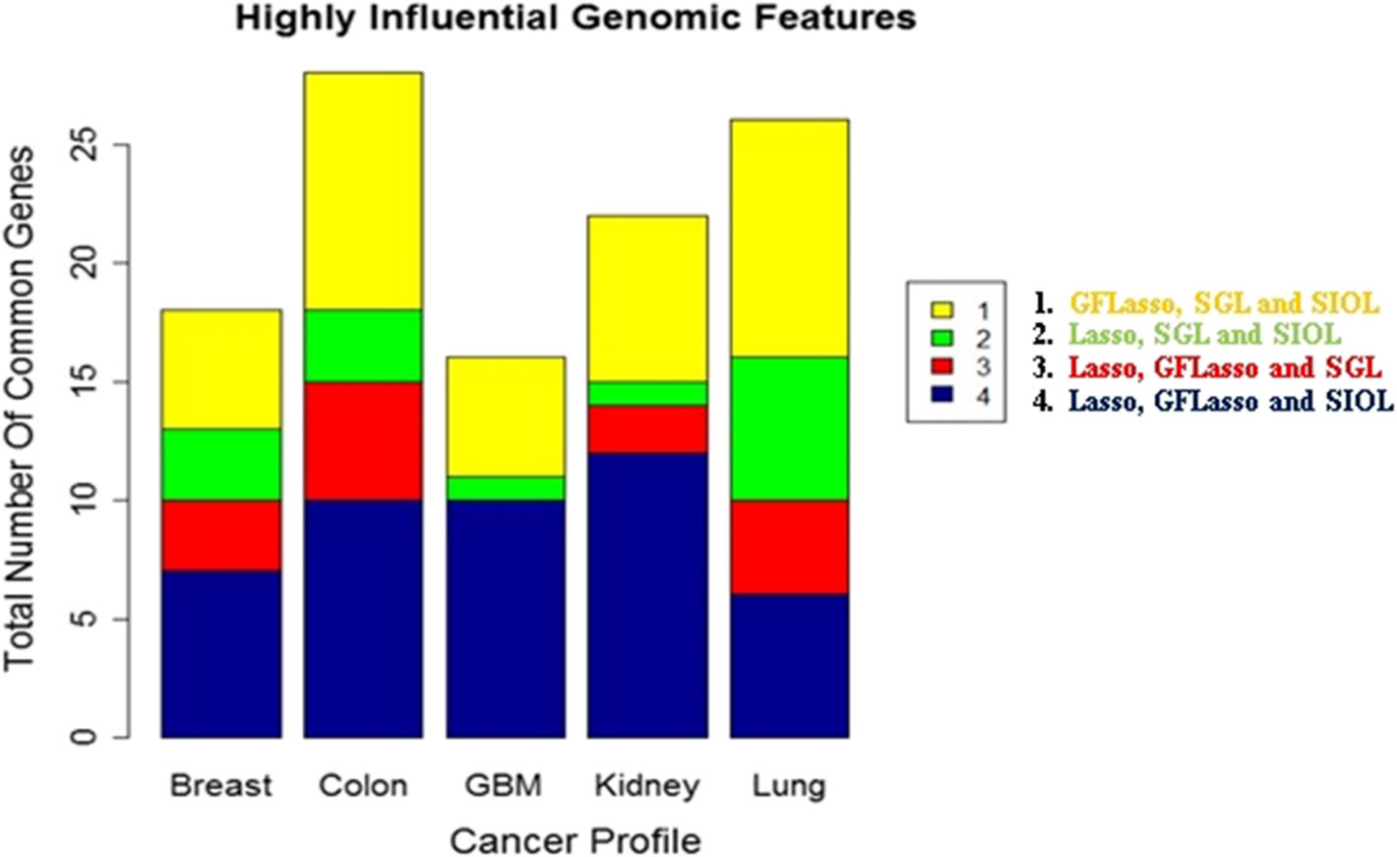
Common methylation features identified by at least three regression methods for each cancer profile

Figure 5 is the summary of the common genomic features identified by at least 3 regression methods. The largest number of identified genomic features was the combination of GFLasso and SIOL, i.e. sets 1 and 4. As observed earlier, considering structural information made the methods operative for identifying the strongest predictor signals of response variables. The total numbers of genomic features identified from the top 200 regression coefficients using three or above regression methods were 21 for the breast, 30 for colon, 20 for GBM, 24 for kidney, and 27 for lung cancer datasets.

Figures 4 and 5 show that the results from different regression methods are very inconsistent. A naïve combination of the results would lead to a biased and inconsistent study. We also observed that selecting the top 50,100, or 150 regression coefficients showed a common trend of 0 (zero) common genomic features identified by all four regression methods (see Additional file 1). Figure 4 shows that the common genes identified by all regression methods from the top 200 regression coefficients were negligible, such as 3, 2, 4, 2, and 1 for the breast, colon, GBM, kidney, and lung cancer data sets, respectively. Therefore, rather than selecting a single regression method, we integrated the results obtained using various regression methods.

### Integrative regression network

#### Permutation scheme to select significant pairs in regression networks

Regression coefficients measure the association strength of genomic features and expression traits. Fusion of these beta coefficients using similarity measurement was observed for different cancer profiles. A similar study can be conducted using correlation measurement, but this correlation is highly prone to identifying additional indirect genomic associations, which may redundantly appear across different types of genomics. We measured the affinities or similarities of methylation features (using the beta matrix) and mRNA expression (by transposing the beta matrix). The four affinity matrices from four regression methods were fused using SNF. The final integrative network was constructed with the strongest affined pairs from each network and from those pairs that were acknowledged by all networks (either stronger or weaker affinity value). Additionally, the affinity of each individual regression method versus the final fused network was examined.

**Fig. 6:**
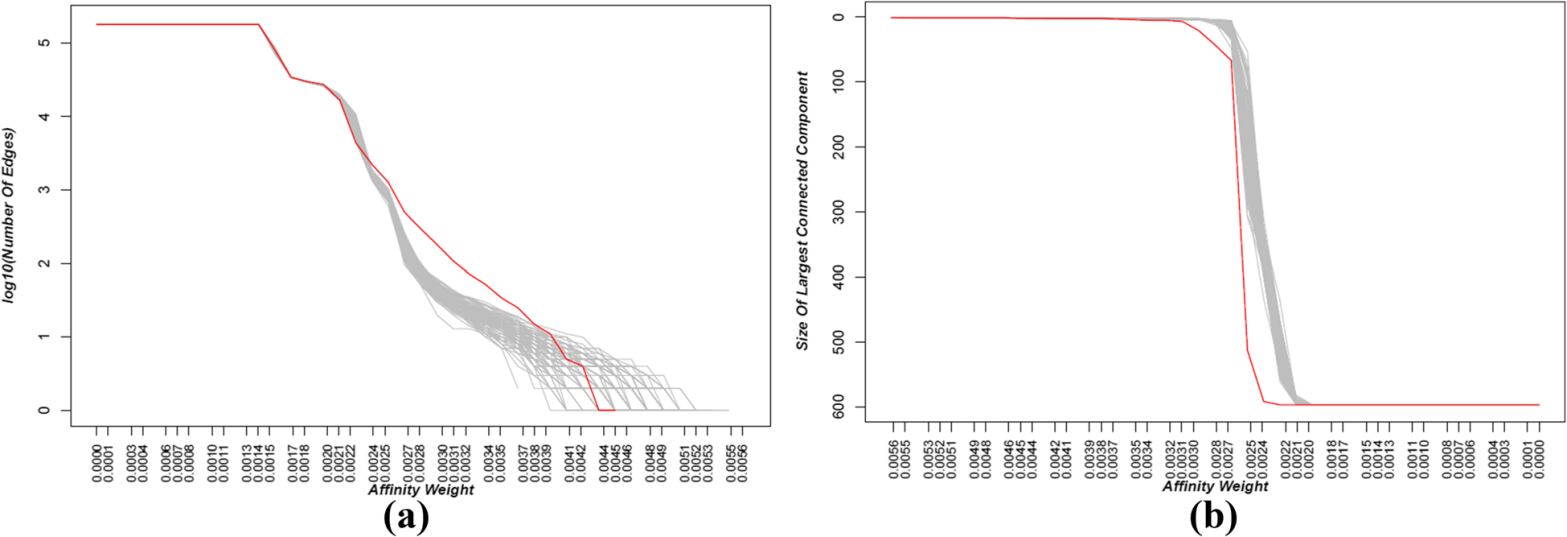
Network properties with varying affinity cutoffs on fused regression network (breast cancer): (**a**) the number of edges and (**b**) the size of largest connected component. Red line shows the result from real data and the gray lines are for the networks of dataset randomly permuted 100 times

We computed the cutoff for identifying significant pairs in the network using equation (8). Figure 6 shows the network properties with respect to varying cutoffs on the fused breast cancer network in comparison with randomly permuted networks. Figure 6a shows the ‘number of edges’ in the real network (red line) and in the network of 100 times randomly permuted datasets (gray lines) and Fig. 6b shows the size of the ‘largest connected component’ in the real network (red line) and the network of 100 times randomly permuted datasets (gray lines). The cutoff obtained using equation (8) for Fig. 6 was 0.0027. Cutoff points were acquired for the affinity matrix of each regression method, and for the fused matrix. For example, the cutoffs found for each affinity matrix and fused network of the colon cancer dataset were 0.2 for GFLasso, 0.225 for Lasso, 0.334 for SGL, 0.175 for SIOL, and for 0.003 fused networks.

Using the cutoff found for all regression methods of all cancer profiles, genomic pairs above the identified cutoffs for both methylation and mRNAs were collected. The overall genomic pairs of methylation and mRNAs are shown in Table 3. It is clear that for each cancer profile, the fused pairs were lower (the total number of genomic feature pairs identified using the four regression methods above the cutoff for breast cancer profile was 19,283 + 5296 + 237 + 1218 = 26,034, but the identified genomic pairs in the fused network was only 238). Hence, the fused network discarded these spurious values and identified stronger affined pairs from each dataset.

**Table 3:** Number of edges after filtering by the identified cutoff in each individual and the fused network

Cancer type	DNA Methylation features					mRNA Expression traits				
	Fused	GFLasso	Lasso	SGL	SIOL	Fused	GFLasso	Lasso	SGL	SIOL
Breast	238	19,283	5296	237	1218	742	182	21,162	811	69
Colon	348	4496	903	288	3260	784	15	18,287	195	104
GBM	584	737	14,925	581	5165	1272	317	22,767	299	480
Kidney	266	4656	3299	1072	2284	1089	44	20,213	4	393
Lung	364	43,752	4497	345	942	696	12	20,589	324	232

#### Regression network properties

Using the selected pairs shown in Table 3 we constructed and examined networks. The constructed network revealed that obtaining a less spurious association was possible only because of combining and integrating the effects of all regression methods. The final fused network showed high confidence, linearity, and modularity. In our experiments, networks were constructed for all cancer profiles, and we studied network properties such as the number of nodes, clustering coefficient, network density, and R^2^ of node degree distribution, among other properties, in Cytoscape [22]. For comparison, the correlation network using methylation and expression data was constructed separately. The edges in this correlation network were those having a *p*-value less than 0.01 divided by the total number of possible pairs in the correlation test. The resulting network properties are summarized in Table 4 for lung cancer, which showed that the fused network was more efficient for identifying a lower number of nodes in association, had a greater number of connected components and exhibited a better R^2^ of node degree distribution. We found similar properties for all other cancer types studied. The R^2^ value for the power-law distribution of all networks (all cancer profiles) showed strong scale freeness [23]. Hence, the proposed technique is effective for identifying crucial cancer-causing genes and discarding unwanted genomic features.

**Table 4:** Network properties of methylation features and mRNA expression trails of Lung cancer profile

Type	Properties	Lasso	GFLasso	SGL	SIOL	Corr.	Fused
Methylation network	Number of nodes	557	418	338	468	552	394
	Network density	0.03	0.50	0.006	0.009	0.12	0.005
	Network diameter	11	5	26	29	7	17
	Clustering coefficient	0.52	0.73	0.28	0.23	0.60	0.14
	Average number of neighbors	16.1	209.3	2.0	4.0	66.8	1.8
	Connected components	16	42	79	50	1	85
	*R* ^*2*^ of node degree distribution	0.52	0.28	0.87	0.40	0.37	0.87
mRNA expression network	Number of nodes	372	17	239	232	276	342
	Network density	0.29	0.09	0.011	0.009	0.04	0.012
	Network diameter	7	3	10	21	9	17
	Clustering coefficient	0.74	0.06	0.29	0.16	0.37	0.27
	Average number of neighbors	110.7	1.4	2.71	2.0	10.3	4.1
	Connected components	2	6	39	46	12	6
	*R* ^*2*^ of node degree distribution	0.13	0.47	0.79	0.98	0.79	0.81

**Fig. 7:**
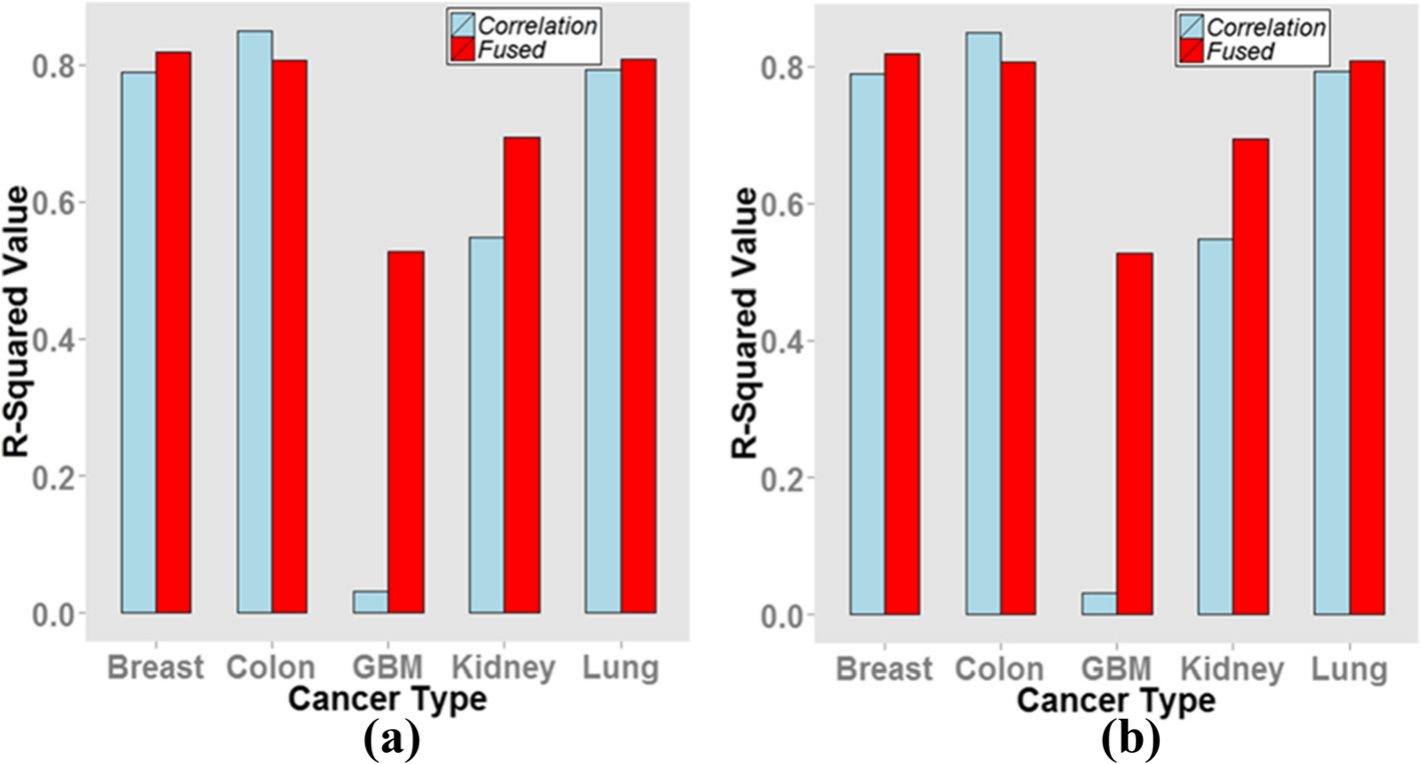
Comparison of *R* ^*2*^ value between the integrative regression network (denoted by “Fused”) and the correlation network. **a** Methylation network (**b**) Gene expression network

Particularly, the fused network showed a better scale-freeness compared with the conventional correlation network. Figure 7 compares the R^2^ values between the integrative regression network denoted by “Fused” and the conventional correlation network measured on the data matrix, not the regression coefficients. For nearly all datasets, except for the kidney dataset in the methylation network and the colon dataset in the mRNA network, the R^2^ measure of the integrated network was significantly larger than that for the correlation measurement.

### Functional characterization of the identified genes

The functional annotation test was performed using Gene Ontology (GO) Biological Process (BP) for the collection of feature genes and expression traits identified in the largest connected component of each network using the tool DAVID [24, 25]. Our studies revealed that (GO: 0042127) regulation of cell proliferation and tyrosine protein kinase and cancer pathway genes, which are overexpressed in high percentages of human cancers, were recognized. The top 5 significantly enriched terms for common methylation features are shown in Table 5. The enriched GO BP terms with the lowest FDR corrected *p*-values were mainly associated with the GBM cancer profile for (GO: 0042127) regulation of cell proliferation, signal, and signal peptide with FDR corrected *p*-values as 1.52E-38, 7.73E-26, and 1.66E-25, respectively. Similarly, functional enrichment studies were performed for expression traits (see Additional file 2). These traits were greatly similar and highly connected in the fused network. The lowest FDR *p*-values were mostly related to chromosomal rearrangement, in nearly all cancer profiles with FDR corrected *p*-values as 5.79E-127 (minimum among all), and hsa05200: pathways in cancer with FDR corrected *p*-values as 4.22E-28.

**Table 5:** Significantly enriched GO BP terms (top 5) for the largest connected component of integrative regression network of methylation features

Cancer type	Category	Term	*N*	*p*-value	FDR
Breast	GOTERM_BP_FAT	GO:0006468 ~ protein amino acid phosphorylation	11	1.32e-07	2.05e-04
	INTERPRO	IPR008266:Tyrosine protein kinase, active site	6	2.35e-07	2.55e-04
	UP_SEQ_FEATURE	binding site:ATP	9	2.81e-07	3.32e-04
	INTERPRO	IPR001245:Tyrosine protein kinase	6	6.25e-07	6.80e-04
	GOTERM_MF_FAT	GO:0004672 ~ protein kinase activity	10	6.90e-07	7.94e-04
Colon	SP_PIR_KEYWORDS	tyrosine-protein kinase	12	1.86e-12	2.33e-09
	INTERPRO	IPR008266:Tyrosine protein kinase, active site	12	1.91e-12	2.50e-09
	INTERPRO	IPR001245:Tyrosine protein kinase	12	1.69e-11	2.21e-08
	SP_PIR_KEYWORDS	signal	40	2.90e-10	3.63e-07
	UP_SEQ_FEATURE	signal peptide	40	3.51e-10	4.94e-07
GBM	GOTERM_BP_FAT	GO:00042127 ~ regulation of cell proliferation	88	8.57e-42	1.52e-38
	SP_PIR_KEYWORDS	signal	128	5.54e-29	7.73e-26
	UP_SEQ_FEATURE	signal peptide	128	1.03e-28	1.66e-25
	GOTERM_BP_FAT	GO:0008284 ~ positive regulation of cell proliferation	54	5.23e-28	9.28e-25
	INTERPRO	IPR001245:Tyrosine protein kinase	27	3.51e-22	5.21e-19
Kidney	GOTERM_BP_FAT	GO:0010033 ~ response to organic substance	19	4.24e-08	6.99e-05
	GOTERM_BP_FAT	GO:0043067 ~ regulation of programmed cell death	18	1.30e-06	0.00214
	GOTERM_BP_FAT	GO:0010941 ~ regulation of cell death	18	1.37e-06	0.00225
	GOTERM_MF_FAT	GO:0032403 ~ protein complex binding	10	1.61e-06	0.00215
	KEGG_PATHWAY	has05200:Pathways in cancer	14	1.65e-06	0.00174
Lung	GOTERM_BP_FAT	GO:0042127 ~ regulation of cell proliferation	25	6.24e-13	1.02e-09
	SP_PIR_KEYWORDS	Proto-oncogene	14	4.78e-12	5.92e-09
	KEGG_PATHWAY	has05200:Pathways in cancer	22	6.02e-12	6.56e-09
	GOTERM_BP_FAT	GO:0007169 ~ transmembrane receptor protein tyrosine kinase signaling pathway	14	1.24e-10	2.02e-07
	GOTERM_BP_FAT	GO:000716 ~ enzyme linked receptor protein signaling pathway	16	2.06e-10	3.37e-07

In enrichment study using gene expression networks, the cancer-related terms were prominently observed, which may be because of the procedure of intersecting expression genes with COSMIC cancer census genes. To cross-verify this result, we randomly selected 30 genes from COSMIC, performed a gene enrichment test, collected the top 5 terms, and repeated the same procedure for 30 iterations. Seven of the top 10 terms were chromosomal rearrangements, but their smallest FDR corrected *p*-values were multiple times larger than the *p*-values obtained from enrichment test of expression traits. A similar trend was identified for other significant terms, such as disease mutation, nucleus, and hsa05200: pathways in cancer, among others.

From the fused networks of methylation features, we collected hub genes that are with highest node degrees (see Additional file 3). Crucial cancer-causing genes were identified from the fused network, including AKT, KRAS, fibroblast growth factor receptors, anaplastic lymphoma kinase, and ERBBs. Previous studied demonstrated that the PI(3)K/AKT pathway is a strong therapeutic target in cell renal cell carcinoma [26]. The KRAS oncogene is mutated in approximately 35–45 % of colorectal cancers and KRAS mutations are considered to be more predominant in pancreatic, thyroid, colorectal, and lung cancers [27, 28]. The anaplastic lymphoma kinase gene was also found to be a relevant term for lung cancer [29]. Overexpression of FEFRs can lead to multiple cancer types and higher levels of fibroblast growth factor receptor was found in prostate, breast, lung, brain, gastric, sarcoma, head and neck, and multiple myeloma cancers [30]. ERBB2 is typically amplified in tumors and overexpressed in breast cancer, and ERBBs are very important in cancer studies [31, 32].

## Discussion and Conclusion

In this study, we presented an integrative regression network by combining the results of different regression methods. Given the highly correlated nature of genomic profiles, the association analysis of conventional correlation tests or multiple regression methods can produce inconsistent results. To address this issue and construct a more reliable association network from genomic profiles, we constructed a regression network by measuring the similarity of regression coefficient vectors in a high-dimensional multivariate multiple output regression setting. The results from different regression methods were further fused using a similarity fusion technique. The fused network facilitated identification of the strongest possible signal and as well as weaker signals, which increased the signal to noise ratio.

The GO enrichment test revealed that the final fused network could identify genes with the lowest FDR corrected *p*-values, and numerous cancer-related features were recognized using the fusion technique. The genes identified using the fusion technique were highly similar behavioral genes for the cancer profile, i.e. if gene *g*_*1*_ and *g*_2_ has nearly the same magnitudes of the regression coefficient and were identified by two or more regression methods or by a single regression method but with a very higher magnitude of similarity, then the SNF allows for the propagation of genes *g*_*1*_ and *g*_*2*_ as nodes in the final network with their affinities (similarities) as edges. Understanding cancer using this process can provide guidance for predicting the prognosis, developing effective therapies, and identifying subtypes [33] of cancer.

We developed an effective method for analyzing genes involved in cancer that integrates results from different regression methods. Although our analysis was done on each of the different cancer types separately, the result can be easily applied to integrate the results from multiple cancer types that can lead to common behavior across cancers. Based on the ease of the fusion technique (SNF), this method can be conveniently adopted to different types of studies in different domains. The properties of SNF such as propagation effect over iterations, robustness against noise and scaling to a large number of genes enables application of this method to many domains.

## Additional files

Additional file 1: Venn Diagram of top 100 regression coefficients. Combining different regression methods results led the study with inconsistent results. To study this we choosed top 200 coefficients, as selecting top 50,100, or 150 regression coefficients showed a common trend of 0 (zero) common genomic features identified by all four regression methods. This file shows the same scenario with top 100 coefficients. (JPEG 135 kb) Additional file 2: mRNA Network Properties. Significantly enriched GO BP terms (top 5) for the largest connected component of integrative regression network of mRNA expressions. (JPEG 282 kb) Additional file 3: Genes With Node Degree 4 and above. This file provides a list of methylation and mRNA genes, those were collected from the highly connected networks (fused) of different cancer profiles. (ZIP 32 kb)
