# Supplementary figures and images for: Integrative regression network for genomic association study
Source: BMC Med Genomics. 2016 Aug 12;9(Suppl 1):31. doi: 10.1186/s12920-016-0192-7 (PMC4989890; doi:10.1186/s12920-016-0192-7)

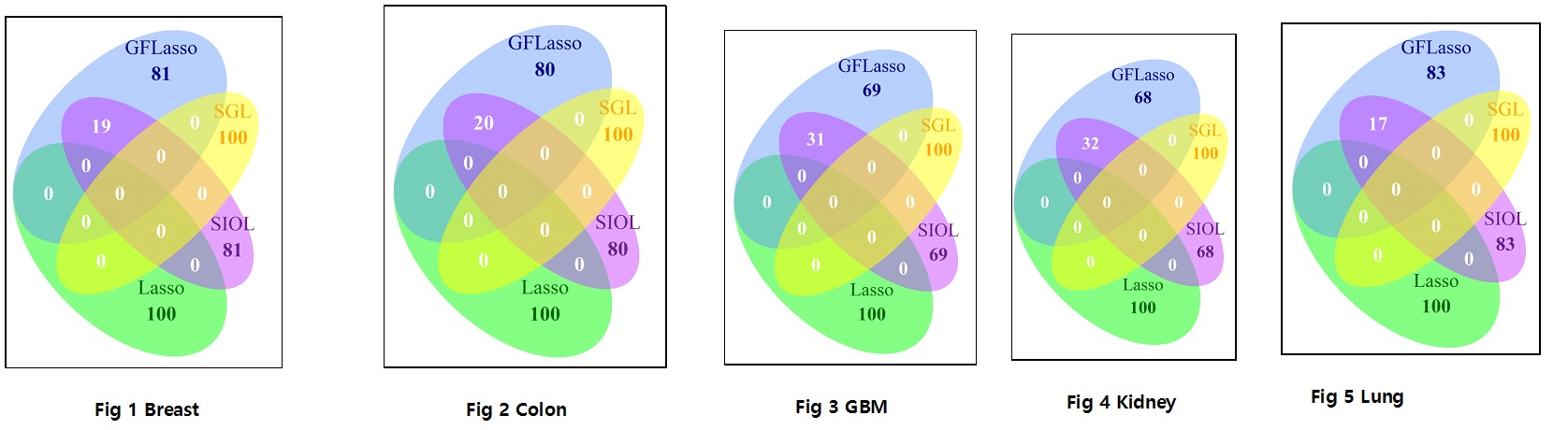

Supplement: Additional file 1: — Venn Diagram of top 100 regression coefficients. Combining different regression methods results led the study with inconsistent results. To study this we choosed top 200 coefficients, as selecting top 50,100, or 150 regression coefficients showed a common trend of 0 (zero) common genomic features identified by all four regression methods. This file shows the same scenario with top 100 coefficients. (JPEG 135 kb) [file 12920_2016_192_MOESM1_ESM.jpeg]

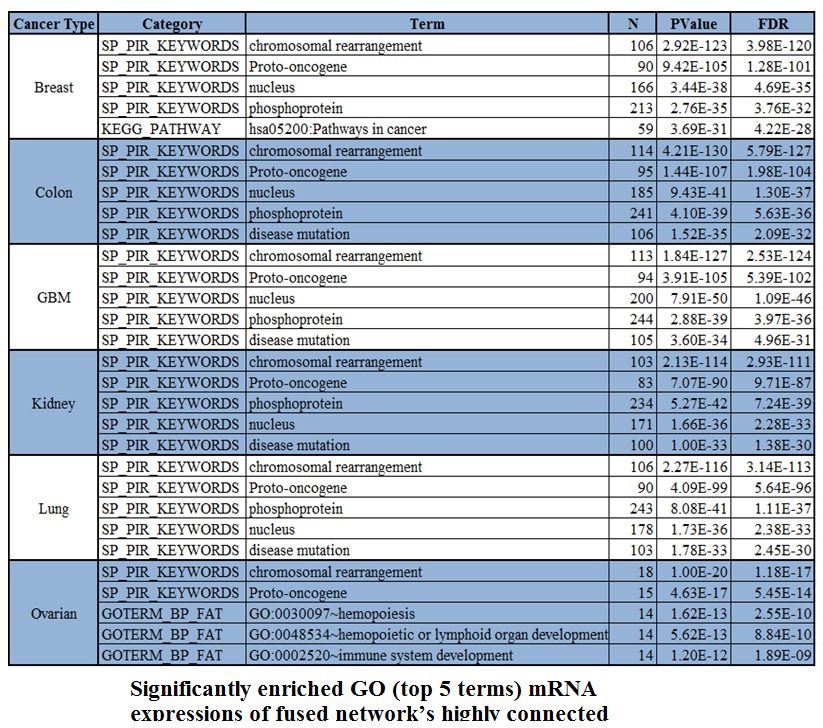

Supplement: Additional file 2: — mRNA Network Properties. Significantly enriched GO BP terms (top 5) for the largest connected component of integrative regression network of mRNA expressions. (JPEG 282 kb) [file 12920_2016_192_MOESM2_ESM.jpeg]
